# Supplementary material for: Are carbon emissions trading and green financial instruments synergistic? -Comprehensive quantitative research based on content analysis
Source: PLoS One. 2024 Mar 7;19(3):e0298601. doi: 10.1371/journal.pone.0298601 (PMC10919601; doi:10.1371/journal.pone.0298601)
Supplement: S1 Data — (ZIP) [file pone.0298601.s001.zip › Data/地级市Z_F工作报告.zip!/╦─┤¿/│╔╢╝╩╨/└┤╫╘╣1⁄2╓┌║┼í╛┬φ┐╦╩2╛▌═°í┐_2018.docx]

各位代表：

现在，我代表市人民政府，向大会报告工作，请予审议，并请市政协委员提出意见。

一、2017年工作回顾及过去五年主要成就

过去一年，在党中央国务院、省委省政府的领导下，按照市委建设全面体现新发展理念的国家中心城市决策部署，我们深入学习贯彻习近平新时代中国特色社会主义思想，牢牢把握稳中求进工作总基调，推动全市经济社会实现平稳健康发展。全年地区生产总值13889.4亿元、增长8.1%，全社会固定资产投资9404.2亿元、增长12.3%，社会消费品零售总额增长11.5%，城乡居民人均可支配收入分别增长8.4%、9.1%，居民消费价格上涨2%，地方一般公共预算收入1275.5亿元、增长11.3%。

（一）保持经济稳定增长。加快构建产业生态圈。启动电子信息、生物医药等13个市级重点产业生态圈建设，加快以66个产业园区为基础的产业功能区建设。深入落实“项目年”工作。京东方第6代AMOLED一期生产线、沃尔沃乘用车整车等189个项目竣工投产，中电子8.6代液晶面板生产线、中车轨道交通装备产业园等146个项目加快建设，格罗方德12英寸晶圆制造、航天通信产业园一期等144个项目开工建设。新签约引进紫光集成电路、药明康德等375个重大项目。全年实际到位内资4812.7亿元、增长15.5%，利用外资实际到位100.4亿美元、增长16.5%。加快发展先进制造业。深入推进“中国制造2025”试点示范城市建设，规模以上工业增加值增长9.0%，工业投资增长33.9%。提升发展现代服务业。服务业增加值增长8.9%。加快建设西部金融中心，金融机构本外币存款余额3.6万亿元、贷款余额2.9万亿元，实现直接融资3328亿元，新增A股上市公司8家、新三板挂牌企业36家。入境游客300万人次，接待游客2.1亿人次，旅游总收入3033.4亿元、增长21.2%。加快建设音乐之都，推进城市音乐厅、露天音乐公园等重大项目建设，音乐产业总收入增长18.4%。优化发展都市现代农业。乡村振兴战略实现良好开局，加快100万亩高标准农田建设，农业增加值增长3.9%。获批创建国家现代农业产业科技创新中心。

（二）扎实推进创新驱动发展战略。大力推进全面创新改革试验。国家第一批复制推广的13项改革经验中，成都占5项。加强协同创新。在蓉高校职务科技成果权属混合所有制改革持续深化，一批科研成果完成确权。新建4个新型产业技术研究院。获批国家知识产权强市创建市，开展知识产权综合管理体制改革试点，成立成都知识产权审判庭。全年新增市场主体46.4万家、增长42%，新增科技型企业2万家、增长55%，高新技术企业达到2473家、增长17.9%，高新技术产业产值增长11.8%。深入推进军民融合创新。创建军民融合供应商管理体系，开工建设涡轮院研发总部、北航西部国际创新港。扎实抓好创新创业。成立新经济发展委员会和新经济发展研究院。新华三成都研究院等项目签约落户。出台人才新政十二条，吸引13万余名本科以上学历人才来蓉落户发展。天府新区、新希望集团获批国家双创示范基地。成功举办2017年创交会，47个国家参会，交易额240.3亿元。组织“菁蓉汇”系列活动188场。建成各类创新创业载体260家、面积1654万平方米。

（三）大力推进重点领域改革。深入推进供给侧结构性改革。扎实开展“三去一降一补”工作，出台产业新政50条，积极落实电、天然气优惠政策，取消停征减免46项行政事业性收费和政府性基金。推进中心城区“11+2”改革。加大住房租赁试点，建立住房租赁公共服务平台，出让租赁住房用地11宗、可建26万平方米。开工建设人才公寓和产业园区配套住房7.8万套，配租配售各类保障性住房1.48万套。深化统筹城乡和农业农村改革。扎实抓好农村集体经营性建设用地入市、农村土地承包经营权有偿退出等改革试点。搭建农村产权交易平台，累计实现交易1.5万多宗、交易额763亿元。开展统筹城乡综合改革示范镇（片）建设。出台积分入户与条件入户“双轨并行”的户籍政策。城镇化率达71.85%。加快推动国资国企改革。实施市属国企“9+2”改革整合转型，开展“压层级、提质量、控风险”专项行动。深化投融资体制改革。开展企业投资项目全流程“承诺制”试点，项目审批时限大幅缩短。稳步推进政府与社会资本合作，累计签约20个PPP项目、总投资1739.5亿元，设立规模为1000亿元的成都发展基金。切实推进商事制度改革，全面实施“先照后证”“多证合一”。完成天府新区和高新区机构重组、全员聘用改革。

（四）持续提升对外开放水平。自贸试验区加快建设。全面启动155项改革试验任务，共新增注册企业1.92万家，注册资本2948.74亿元。国际班列扩容增效。蓉欧快铁新开行成都至莫斯科、布拉格等9条线路，国际班列全年共开行1012列，货值46亿美元、增长254%，位居全国中欧班列第一。航空肉类进境指定口岸获批，实现空港“7×24小时”客运口岸通关、货运口岸预约通关。成都双流机场旅客吞吐量4980万人次、增长8.2%，其中出入境流量539万人次、增长8.7%，位列中西部城市第一；货邮吞吐量64.3万吨、增长5.1%。新开通成都至纽约、奥克兰等5条国际定期直飞客运航线。“走出去”工作稳步推进。成都企业在“一带一路”沿线国家在建和筹建项目共计166个，涉及金额超过100亿美元。进出口贸易总额3941.8亿元、增长45.4%。对外交流合作持续深化。成功举办联合国世界旅游组织第22届大会等重大国际活动，成为“金砖”国家电影节在中国的永久举办地。中德、中法、中意、中韩、新川等国别合作园区加快建设，中国—欧洲中心投入使用。新增拉普拉塔、马普托2个国际友城和曼谷、瓦伦西亚等12个友好合作关系城市。

（五）加快提升城市功能品质。城市空间功能格局进一步优化。积极开展总规修编改革试点，编制《成都市城市总体规划（2016-2035年）》，统筹推进“东进、南拓、西控、北改、中优”。综合交通通信枢纽功能进一步增强。天府国际机场主体工程及配套设施加快建设，双流国际机场扩能改造顺利推进。《成都铁路枢纽规划（2016-2030年）》获批，西成客专、成昆铁路成都至峨眉段扩能改造建成投用，火车北站扩能改造、成蒲铁路等项目加快推进。成德动车加密开行。轨道交通加速成网，先后开通运营地铁4号线二期、10号线一期和7号线。编制完成《成都市域高快速路网体系规划》《成都市慢行交通系统规划》，成安渝高速、三绕高速南段建成通车，天府国际机场高速、成彭高速扩能改造、三绕高速西段和东段加快推进。新增非机动车道1130公里，打通断头路90条，完成村组道路建设1600公里。启动“互联网+城市”行动，“宽带中国”示范城市加快建设，成为国内首批5G试点城市。城市基础配套功能进一步强化。启动中心城区公共服务设施“三年攻坚”行动，编制“818” 城市十五分钟基本公共服务设施规划，开工建设450个项目，建成276个。实施“科学治堵”，深化公交都市建设，率先出台鼓励规范共享单车、新能源汽车分时租赁行业发展政策。实施城乡厕所革命攻坚行动，新建、改建公厕336座，被评为全国厕所革命优秀城市。持续开展城乡环境专项整治，新打造特色街区20条，完成城市棚户区改造1.17万户、老旧院落改造920个、城中村改造7427户。

（六）全力推动绿色低碳发展。推进国家低碳城市试点建设。绿色低碳能源体系加快构建。推动工业企业“煤改气”“煤改电”，推广运输工具油改气，加快建设充电站（桩）设施，新增电动汽车2.1万台。认真开展中央、省环保督察迎检工作，加强督察整改。污染防治“三大战役”成效明显。实施“治霾十条”和大气“650”工程，淘汰燃煤锅炉889台、黄标车2.39万辆，完成建成区和场镇餐饮店“煤改气”“煤改电”3968家，对1577个建设工程项目进行扬尘在线视频监测，清理关闭“散乱污”企业14148家，空气优良天数235天，比上年增加21天，是2013年实行环境空气质量新标准以来达到的最长天数，空气优良率在全国74个重点城市排名比上年上升7位，PM10、PM2.5年均浓度同比下降13.7%、10.2%，均创历史最好水平，增强了我们打赢蓝天保卫战的信心。实施“治水十条”和水“626”工程，严格落实河长制，完成城市建成区53段黑臭水体综合整治，岷江等流域水质逐步向好。实施“治土十条”和土壤“620”工程，启动全市土壤污染源详查工作，开展了重点企业土壤调查和空间位置遥感核实。城市碳汇能力体系加快建设。开展全域增绿行动，实施“增绿十条”，打响城区绿化“春季秋季战役”，启动并加快建设龙泉山城市森林公园、天府绿道，新增绿地809万平方米。开展“花重锦官”增花添彩工作，市花芙蓉花总量翻了一番。开展节能低碳产品认证，绿色低碳消费体系不断完善。加大机关事务管理标准化和公共机构节能减排工作力度。入选全国首批装配式建筑示范城市。国际城市可持续发展高层论坛在蓉举办，发布《成都宣言》，被列为联合国人居署“可持续发展试点示范城市”。

（七）创新落实“放管服”工作。进一步精简行政审批事项。取消行政许可18项，“全链条”下放关联、相近类别事项，行政许可标准化建设加快推进，村（社区）证明事项从313项大幅缩减到15项，获得国务院肯定。强化事中事后监管。在19个区（市）县设置市场和质量监督管理局，全面推行“双随机、一公开”监管。大力推进网络理政。全年累计收到群众来电来信236.3万件，回复办理率99.2%、诉求解决率84.1%、群众满意度86.7%。12345市长公开电话荣获“百分百接通”奖和“先锋”奖，成都网络理政创新实践在第五届中国民生发展论坛上被评为“2017民生示范工程”第一名。41个部门单位727个审批服务事项实现网上办理，开通率达97.6%。开展政务公开标准化、规范化试点，主动通过全市网络平台公开各类政务信息400余万条，行政规章、规范性文件公开率100%。规划构建“云、网、端、数”四位一体的政务云平台，积极利用大数据推进科学决策。深入推进“两集中、两到位”，市级行政审批99.8%入驻并授权给市政府政务中心，继续在多领域试点“一窗式”服务，推行“仅跑一次”改革。在11个部门和系统开展错时延时工作制试点，全市政务服务系统错时延时服务窗口办件总量36.6万件，让企业和群众办事更省心、更撇脱。

（八）持续保障和改善民生。强力推进脱贫攻坚。简阳85个省定贫困村、29878人全部实现脱贫退出，新建和改造贫困户住房8380户。共投入援助资金4.27亿元、选派532名干部对口支援甘孜、阿坝19个深度贫困县。积极支持九寨沟灾后恢复重建。启动国家首批健康城市试点建设。城市公立医院全部取消药品加成，组建医联体82个，中医药医疗服务体系不断健全。孕产妇死亡率4.66/10万，为历史新低。就业社保工作扎实推进。城镇新增就业27.6万人，年末城镇登记失业率3.15%，动态消除“零就业”家庭，“散乱污”企业本市户籍失业人员再就业率达95.96%。切实加大对城市一线管理服务人员关爱力度，修建环卫工人作息房300余间，为16519名环卫工人进行免费体检，为公交司机提供公租房，让他们感受到城市的关爱和温度。社会救助体系不断完善，助残惠残“三大工程”服务264万项次。新增养老服务床位1.4万张，开展长期照护保险试点。教育事业优先发展。新建、改扩建中小学校21所、39所，建成公办幼儿园160所，新增学位9万个。投入1.6亿元改善农村义务教育学生营养。出台全国首部社区教育地方法规。高等教育质量稳步提升，8所在蓉高校入选国家“双一流”建设计划，成立中国—东盟艺术学院。大力发展文化体育事业。成都演艺中心投入运营。成功举办第六届中国成都国际非遗节、第五届中国网络视听大会、国际诗歌周等品牌节会，再启“蓉城之秋”成都国际音乐季，开展文化四季风等文化惠民活动，连续四届蝉联全国文明城市荣誉称号。入选世界文化名城论坛第34个会员。举办首届成都国际马拉松赛等19项国际赛事，新建公益性足球场139块。国防动员能力增强。积极开展防灾减灾工作。食品药品安全形势稳定向好，全年未发生重特大安全生产事故，安全生产事故起数下降20.95%、死亡人数下降13.57%。扎实推进“七五”普法，荣获全国“法治政府建设典范城市”等称号。平安成都建设不断深化，城市运行安全平稳有序，再度荣获全国综治最高奖“长安杯”，让城市更安宁、市民更舒心、生活更安逸。

市政府承诺的年度十大民生实事全面完成。

各位代表，2017年各项目标任务全面完成，标志着本届政府工作目标胜利实现。过去五年是成都发展史上不平凡的五年，我们经受种种考验、克服重重困难，推动城市实现跨越发展，迈过人均地区生产总值过万美元和地区生产总值过万亿元两大台阶，综合实力居省会城市前列。

——这五年，产业转型升级实现新发展。英特尔“骏马”芯片封测、东风神龙汽车等一批重大项目建成投产。先进制造业和战略性新兴产业收入占工业总收入比重达59%。形成电子信息、汽车制造、装备制造、食品饮料、旅游、金融、物流等7个千亿级产业集群和高新区、经开区2个千亿园区。服务业动力培育取得新成效，都市现代农业和新农村建设走在全国前列。三次产业结构为3.6︰43.2︰53.2，增加值分别年均增长3.8%、8.7%、8.9%，产业融合发展不断催生新业态，城市产业竞争力大幅提升。

——这五年，经济发展集聚新动能。国家级工程实验室、国家企业技术中心分别增加了2家、13家。获得国家级科技奖励113项，引进诺贝尔奖获奖团队6个。四川航天云网、翼龙无人机等军民融合重大项目建成。四川大学、郫都区等5家单位获批国家双创示范基地。全社会研发投入年均增长13.5%，每万人发明专利拥有量年均增长18%。新增科技型企业5.25万家。各类市场主体超过138.5万户、年均增长31%。

——这五年，城市功能品质实现新提升。实施“东进、南拓、西控、北改、中优”，城市空间加快优化，重塑产业经济地理，中心城区形成“11+2”格局。天府新区获批国家级新区，核心区承载功能加快构建。以北改工程为龙头，中心城区加快有机更新。全面完成简阳代管，双流、郫县撤县设区。圆满完成国家统筹城乡综合配套改革各项任务，“四好农村路”建设成绩显著。天府国际机场开工建设。成渝、西成、成绵乐客专开通运营。西部高速公路主枢纽初步建成。城市轨道交通加速成网，运营里程达到179公里，轨道交通占公共交通出行分担比率提升到35%。慢行交通系统加快修复提升，自行车以共享形式回归城市。成都博物馆、西部博览城等一批重大城市功能设施建成投用。

——这五年，对外开放合作迈出新步伐。驻蓉领事机构增至16个，国际友城和友好合作关系城市达到85个，世界500强企业落户281家。国际（地区）航线达到104条，航空“第四城”地位更加巩固。蓉欧快铁连接境外14个、境内12个城市，成为中欧班列中开行线路和数量最多、运行最为稳定的班列。成功承办《财富》全球论坛、世界华商大会、G20财长和央行行长会议等国际性会议。进出口贸易保持较高增长势头，与“一带一路”沿线国家（地区）经贸往来呈加速态势。

——这五年，生态文明建设实现新突破。超额完成“十二五”总量减排任务，万元地区生产总值能耗逐年降低。全面打响大气、水、土壤污染防治“三大战役”。2017年与2013年相比，PM10、PM2.5浓度分别下降40%、42.3%，空气优良天数增加103天、达到235天。完成城市建成区黑臭河渠综合治理。土壤环境质量保持稳定。龙泉山、龙门山生态植被恢复等重大生态工程成效显著。森林覆盖率增至39.1%、蓄积量达3367万立方米。绿色低碳发展制度体系初步构建。成功创建国家生态市和国家低碳试点市。

——这五年，保障改善民生获得新进展。财政一般公共预算民生支出占比平均达66.8%。城镇新增就业132万人，城乡居民人均可支配收入年均分别增长8.5%、11%，分别达3.89万元、2.03万元。城乡统一的社保体系全面形成。养老服务床位达11.4万张。人均期望寿命达到79.89岁。建成各类保障性住房8.4万套，解决近20万群众的住房困难。教育综合改革成绩突出。成为全国首批公共文化服务体系示范区。全面消除绝对贫困，完成简阳脱贫攻坚，积极对口支援甘孜、阿坝。圆满完成“4·20”芦山强烈地震灾后恢复重建和对口援建雅安任务。连续七届荣获“全国双拥模范城市”。蝉联“中国最具幸福感城市”榜首。

——这五年，政府自身建设取得新成效。严格落实中央八项规定和省市相关规定精神。深入开展党的群众路线教育实践活动和“三严三实”专题教育，推进“两学一做”学习教育常态化制度化，干部“走基层”广泛开展。党风廉政建设和反腐败斗争深入推进，依法行政、廉洁从政的政治生态不断向好。“放管服”改革持续深化，政府惠企利民能力明显增强，群众对政府的满意度持续提升。

国家安全、防灾减灾、应急管理、消防、人防工作切实加强，审计、统计、档案、保密、气象、参事、地方志、哲学社会科学等工作顺利推进，民族宗教、港澳台、妇女儿童、青少年、老龄、慈善等工作成效明显。

各位代表，这些成绩的取得，是党中央国务院、省委省政府和市委坚强领导的结果，是市人大、市政协监督支持的结果，是全市干部群众共同努力的结果。在此，我代表市人民政府，向全市人民，向人大代表、政协委员、各民主党派、工商联、无党派人士、各人民团体，向中央和省驻蓉单位，向所有关心支持成都发展的同志们及各界人士，表示衷心的感谢！向人民解放军指战员、武警及消防部队官兵、政法干警和民兵预备役人员，表示崇高的敬意！向港澳台同胞、广大侨胞和海内外朋友们，表示诚挚的谢意！

在看到成绩的同时，我们也清醒认识到，全市经济社会发展还存在不平衡不充分的问题，工作中还有许多不足。实体经济转型升级步伐还需加快，各类市场主体活力还未充分激发，产业现代化水平总体不高；创新驱动能力还有待加强，新经济新动能还需加大培育力度；对外开放的广度深度还不够，在更宽领域、更高层次参与全球竞争的能力还不强，城市国际化进程还需加快；资源环境约束趋紧，生态文明建设需进一步提速，特别是大气、水、土壤污染防治攻坚任务繁重；群众在教育、医疗、居住、出行、养老、文体需求等方面还存在不少难题，城市运行安全保障和精细化管理尚需进一步加强；少数干部专业化水平不高，运用互联网和现代技术开展工作的本领还需加强，抓落实强担当能力需要提升、作风需要改进。对这些问题，我们将切实采取有力措施加以解决。

二、今后五年工作

中国特色社会主义进入了新时代。新思想引领新时代，新使命开启新征程。成都已站在了新的历史起点，多重历史机遇叠加，前所未有、千载难逢，昭示着成都的美好前景，召唤我们开拓创新、乘势而上，在新时代创造新的成都业绩！

我们要深入贯彻党的十九大精神和习近平新时代中国特色社会主义思想，坚决落实习近平总书记来川视察重要讲话精神，统筹推进“五位一体”总体布局，协调推进“四个全面”战略布局，坚定不移贯彻新发展理念，坚持稳中求进工作总基调，全面落实党中央国务院、省委省政府和市委决策部署，按照新时代成都“三步走”战略目标，加快构建现代化经济体系，实现经济高质量发展，建成全面体现新发展理念的城市。

我们要重点完成五大任务：一是坚持创新发展，筑牢现代化经济体系的战略支撑。深入实施创新驱动发展战略，突出产业关键技术和共性技术创新，推动经济发展质量变革、效率变革、动力变革，以创新为支撑的现代化经济体系基本形成。二是坚持协调发展，提升城市整体发展层次水平。落实主体功能区战略，实施“东进、南拓、西控、北改、中优”，重塑产业经济地理。强化国家级天府新区战略定位，加快打造城市重要极核。落实乡村振兴战略，城乡融合一体发展水平不断提升。科学制定城市中长期人口规划，实现人口、资源、环境协调发展。三是坚持绿色发展，形成人与自然和谐发展新格局。建立绿色能源、生产、消费体系，推动经济社会逐步实现绿色低碳转型。继续实施“三治一增”，打好大气、水、土壤等污染防治“三大战役”，实施一批生态保护和碳汇工程，努力还自然以宁静、和谐、美丽，城市可持续发展的根基不断夯实。四是坚持开放发展，厚植对外合作竞争新优势。深度融入国家“一带一路”和长江经济带建设，不断提高双向开放水平，加快打造国际门户枢纽城市，城市国际影响力大幅提升。五是坚持共享发展，增强城乡居民获得感幸福感。坚持人民城市为人民，打造绿色宜人的生态环境、开放包容的人文环境、舒适安逸的生活环境、优越便利的创业环境、廉洁高效的政务环境。提供更多更优的公共服务供给，构建更好民生保障体系，不断促进有感发展，满足人民群众对美好生活的需要。

三、2018年工作安排

2018年，是全面贯彻落实党的十九大精神的开局之年，是改革开放40周年。做好今年工作的基本思路是：要以习近平新时代中国特色社会主义思想为指导，全面贯彻落实党的十九大和中央经济工作会议、习近平总书记来川视察重要讲话精神，落实省第十一次党代会、省委十一届二次全会及市第十三次党代会、市委十三届二次全会的部署，坚持“党是领导一切的”，坚持稳中求进工作总基调，坚持新发展理念，坚持以人民为中心的发展思想，坚持以供给侧结构性改革为主线，坚持加快构建现代化经济体系，紧扣社会主要矛盾变化，围绕市委总体安排，把今年确定为“全面落实年”，统筹推进稳增长、促改革、调结构、惠民生、防风险各项工作，推动质量变革、效率变革、动力变革，打好防范化解重大风险、精准脱贫、污染防治的攻坚战，落实好全省“四项重点工程”，加强和改善民生，促进经济社会持续健康发展。

今年发展的主要预期目标是：地区生产总值增长7.5%左右，全社会固定资产投资增长8%，社会消费品零售总额增长10%，进出口总额增长8%，地方一般公共预算收入增速与经济增速同步，城乡居民人均可支配收入分别增长8%、9%，全社会研发投入增长12%，万元地区生产总值能耗、主要污染物排放进一步降低，居民消费价格上涨控制在3%左右，城镇登记失业率控制在4%以内。重点抓好以下九个方面工作：

（一）夯实实体经济落实高质量发展。坚持把经济的着力点放在实体经济上，把提高供给质量作为主攻方向，继续落实省委省政府“项目年”工作，加大培育创新生态链，大力推进66个产业园区建设，着力构建产业生态圈，努力在中高速增长中推动经济高质量转向。

一是加快发展先进制造业。加快推进“中国制造2025”试点示范城市建设，积极争创国家级示范区。深化“互联网+先进制造业”，大力发展新一代信息技术、生物医药、轨道交通等战略性新兴产业，提升传统工业智能化、信息化水平，加快建筑工业化进程，培育壮大产业集群。力争工业投资增长10%以上，规模以上工业增加值增长8.5%以上。抓好工业项目建设，促进通威高效晶硅电池、科伦生物医药产业基地等项目加快建设，支持极米科技、腾盾科技等创新企业快速发展。推进产业功能区差异化、特色化发展，制定分行业的项目准入标准，加快提升产业层次，推动产业融合发展。

二是大力发展现代服务业。 深入实施“商圈提升”行动，建设37个服务业集聚区、39个现代商圈和100条特色街区。服务业增加值增长8.5%以上。加快发展研发设计、咨询、检验检测、现代物流等生产性服务业，深入推进国家供应链体系建设综合试点和国家现代物流创新发展城市试点，深入实施城市共同配送试点。大力发展商业零售、文化服务、旅游休闲、餐饮服务、医疗健康服务、养老服务、教育服务、体育服务、居民和家庭服务等生活性服务业，加快中法合作·成都国际智慧养老服务示范社区项目试点。发展智慧服务、体验服务等服务业新形态。加快建设世界旅游名城。大力发展全域旅游，加快推进航空大世界等项目建设，促进青城山、西岭雪山—花水湾创建国家级旅游度假区，支持安仁古镇、平乐古镇—天台山创建国家5A级旅游景区，用好大熊猫、美食、休闲、绿道等名片，推出更多创意经典的“成都礼物”。设立旅游境外推广体验中心，举办成都国际旅游展等节会活动，力争入境游客330万人次，游客总数2.18亿人次，旅游总收入突破3600亿元。

三是大力发展都市现代农业。把发展现代农业作为实施乡村振兴战略的重中之重。加快天府农博园、崇州优质粮油产业园等重点产业园建设，积极争创国家农业高新技术产业开发区，加快建设现代农业科技创新中心。培育一批大企业大集团和总部型企业，积极培育家庭农场和农民合作社。实施“农业+旅游”“农业+康养”“农业+互联网”“农业+会展”行动，加大培育农产品和食品品牌，加强食品行业老字号的保护和传承，用品牌引领农业高质量发展，让农业成为有奔头的产业，让农民成为有吸引力的职业。力争农产品加工产值达到1500亿元、休闲农业和乡村旅游总收入达到380亿元。实施优势特色农业提质增效计划，大力推进农田水利基本建设，建好100万亩高标准农田。推进崇州市、蒲江县全国农村产业融合试点示范县建设。实现农业增加值增长3%。

四是增强金融服务实体经济能力。加快西部金融中心建设，推动“1+5+N”金融产业聚集区建设，大力引进法人金融机构和区域性金融总部。完善企业全生命周期创业投资体系，强化创投融资服务，搭建差异化产融对接平台，更好满足各类市场主体的融资需求。用好多层次资本市场，实施企业上市倍增行动计划，力争新增上市公司8家，新三板挂牌企业40家。着力发展“科技金融”“农村金融”“消费金融”“供应链金融”，力争2018年新增信贷投放超过3000亿元，直接融资规模超过3500亿元。积极发展碳金融，加快构建以直接投融资、银行贷款、碳指标交易、碳期权期货等为支撑的碳金融体系。发挥政府基金引领作用，带动社会资本投入我市重大项目。以天府国际基金小镇为载体，打造“募、投、管、退”基金业全产业链生态系统。建立健全地方金融风险监管协同机制，规范地方金融秩序。加大打击和处置非法集资力度，抓好互联网金融风险专项整治和交易场所清理整顿，守住不发生区域性金融风险的底线。

（二）持续加快发展动能转换。深入实施创新驱动发展战略，扎实推进全面创新改革，着力提高全要素生产率，加快培育创新生态链，汇集改革动力、激发人才活力，推动转型发展、高质发展。

一是大力发展新经济培育新动能。重点发展数字经济、智能经济、绿色经济、创意经济、流量经济、共享经济等新经济形态，培育智慧城市建设、现代供应链等七大应用场景。建立新经济天使投资基金。实施新经济企业梯度培育计划，实施科技型、创新型中小企业培育工程，形成“千帆竞发”的生动局面，力争新增新经济企业5000家以上，培育独角兽企业2家。打造一批新经济核心区和集聚发展区。推动数字经济和实体经济融合发展，促进物联网、大数据、人工智能、区块链、虚拟现实等新兴技术的发展与应用，积极发现、支持、培育各类新经济增长点。

二是深入推进政产学研用协同创新。实施蓉城科技聚变计划，强化基础研究和应用基础研究，全力争创综合性国家科学中心，积极争取国家重点实验室、国家工程研究中心等重大科研平台布局落地。建立校院企地共建共享机制，推动创新资源要素向产业功能区聚集和优化配置，共同开展科技攻关、成果转化和企业培育。加快科技成果向现实生产力转化，深化高校院所职务科技成果权属混合所有制改革。落实强化首台套政策。强化知识产权管理、保护和运用，建设成都知识产权交易中心、版权交易中心和高价值专利培育中心，探索知识产权证券化。

三是持续推进军民融合创新。探索“民参军”机制创新和“军转民”开放创新，积极争创国家军民融合创新示范区，打造共通共用共建共享的军民融合示范平台，启动建设军民融合创新中心和产业园。加快激光、核动力、航空动力等7个省级军民融合产业基地建设，加快成飞公司航空整机、成都信息安全产业园等军民融合重点项目建设。

四是加快建设国家自主创新示范区。编制印发《成都国家自主创新示范区发展规划纲要》，推动出台《成都国家自主创新示范区条例》。着力打造电子信息、新经济、生物等三大产业生态圈，加大领军企业培育力度，加快中国—欧洲中心、新川创新科技园等创新创业中心建设，努力建设成为创新驱动发展引领区、高端产业集聚区、开放创新示范区和西部地区发展新的增长极。

五是扎实抓好大众创业、万众创新。加快国家级“双创”示范基地建设，支持郫都区菁蓉镇、高新区菁蓉国际广场、天府新区国际菁蓉中心建设，加快建设环高校知识经济圈，打造更多高能级众创空间和科技孵化器。大力发展研发设计、技术交易、创业投资、技术评估等创新服务平台，建立健全“创业天府”云孵化体系。深入落实人才新政十二条，实施“全球顶级科技园区合伙人计划”，推进“蓉漂计划”等人才工程，完善人才扶持和服务体系，建设海外人才离岸创新创业基地和人力资源服务产业园，构建人力资本协同体系，引进高端人才200名以上，吸引更多人才到成都创新创业。深化“菁蓉汇”品牌活动，高水平办好2018年双创周、创交会等活动。

（三）继续深化重点领域改革。横向讲协调、纵向讲渐进，多搞“小切口”，点线面结合推动改革，减少震荡、改出实效。

一是深化供给侧结构性改革。紧扣中央“三去一降一补”“破、立、降”要求和省委省政府“一提一创一培”重点任务，优化存量资源配置，扩大优质增量供给。以产出为导向配置土地资源，发挥项目投资对供给的关键性作用，实施重点项目1000个、年度投资4000亿元以上。落实产业新政50条，降低企业用能、物流等要素成本，取消“五路一桥”收费，降低口岸操作成本。扩大民间投资，大力支持非公经济发展，实施“蓉归故里”计划，支持发展校友经济。加快建设“品质成都”，提升“成都标准”，扩大“成都制造”“成都创造”“成都服务”影响力。弘扬企业家精神和工匠精神，营造劳动光荣的社会风尚和精益求精的敬业风气。

二是深化住房制度改革。坚持“房子是用来住的、不是用来炒的”定位，完善房地产市场调控制度体系。推进住房租赁试点改革，发挥国有住房租赁公司示范带动作用，积极规范培育壮大租赁市场。完善租售并举的住房制度，推进实施保障型公共租赁住房租赁补贴制度，不断完善公积金缴存使用制度，全面推开条件入户和积分入户，支持市民住房刚性需求。试点建设共有产权住宅，大力实施人才安居工程。

三是深化国资国企改革。深入推进国有资本优化布局，以规范法人治理结构和深化“三项制度”改革为重点，建立健全现代企业制度。以管资本为方向，改革完善国有资本授权经营体系，建立健全以效率为导向的国资经营评价体系和监督管理制度。积极开展市属国企“降负债、降成本、提效益、提能力”行动，大力实施“人才强企”战略。力争市属国企资产证券化率达12%以上，新增上市或挂牌市属国企4户以上。

四是深化财税和投融资体制改革。全力推动以绩效为导向的财政预算制度改革，系统谋划财源建设，加快转变财政投入方式，推动建立可持续的现代财政制度。加强政府债务限额管理，严格限定举债规模，规范举债程序和资金用途，切实防范政府债务风险。做优做强国有投资平台，更好保障重大项目建设。积极争取中国政企合作投资基金的支持和引导，大力推动政府与社会资本深度合作。

五是深化商事制度改革。加快推进“多证合一”等改革，推进工商登记全程电子化和电子营业执照广泛应用。在自由贸易试验区实行“企业住所申报承诺制+负面清单管理”，放宽企业住所登记条件。产业园区全面实施企业投资项目承诺制试点，完善投资项目“三个清单”管理制度。加快社会信用体系示范城市建设，推行统一社会信用代码，建立联动响应、联合惩戒机制。

（四）扎实推进乡村振兴战略。坚持农业农村优先发展，把生活富裕作为实施乡村振兴战略的中心任务，按照产业兴旺、生态宜居、乡风文明、治理有效、生活富裕的总要求，加大城乡融合发展力度。

一是持续深化农业农村改革。科学制定乡村振兴战略规划。深化农村土地制度改革，落实第二轮土地承包到期后再延长三十年政策，推进承包地“三权”分置，加快农村“三地”改革。深化农村集体产权制度改革，做实农村集体经济，抓好集体经营性建设用地入市试点，完善土地出让、租赁、入股和增值收益合理分配机制。继续推进建设用地指标增减挂钩试点。推广“农业共营制”，推进农村土地有序流转，力争土地适度规模经营率达到65%。探索“管委会+平台公司”管理新模式。积极稳妥推进乡镇整合、镇改街道、合村并组、村改居工作。

二是提升农村资源要素供给质效。建立资本下乡激励规范机制，完善“农贷通”金融综合服务平台。鼓励支持区（市）县与高校院所、企业共建农业技术试验推广基地，培育引进农业专业技术人才和专业机构。实施农民工返乡创业行动计划，创建国家级返乡创业示范园区。深化新型职业农民培育试点，大规模开展农村实用技能免费培训。支持农产品电商平台和乡村电商服务站点建设，加快农业大数据中心、物联网云平台等农村信息工程建设。

三是加快美丽乡村建设。制定乡村振兴战略空间发展规划、示范走廊规划。积极开展“三美四好”示范村建设，深入开展“四好农村路”建设。加快特色镇（街区）建设和川西林盘保护修复。积极打造“特色镇+平台+田园综合体”模式，推进大地景观再造，加快都江堰精华灌区大地景观、航空沿线旅游景观等重大项目实施。推进乡村社会化服务建设，整治提升农村人居环境，建设美丽宜居乡村。

（五）优化完善城市功能品质。围绕城市空间结构调整和产业经济地理重塑，深入实施主体功能区战略，统筹推进“东进、南拓、西控、北改、中优”，系统推进城市有机更新，高标准建设“三城三都”，加快形成更有效率、更可持续的城市综合承载体系。

一是加快天府新区建设步伐。充分发挥天府新区在“一带一路”建设和长江经济带发展的重要节点作用，围绕建设西部地区最具活力的新兴增长极、内陆开放经济高地、宜业宜商宜居的国际化现代化新城的定位，突出公园城市的特点，把生态价值考虑进去，用战略眼光和全球视野谋划新区未来发展。高标准推进天府新区总规修编。统筹基础设施成网配套和高端产业集聚发展，完善交通能源、公共服务、生态绿地等城市功能。加快鹿溪智谷、成都科学城、天府中心等重点区域开发建设，促进中国移动（成都）5G联创产研院、西南地球科学创新中心等项目开工建设，切实发挥天府新区对全省全市经济社会发展的辐射带动作用。

二是大力推进天府国际机场和国际空港新城建设。围绕打造一流的国家级国际航空枢纽和内陆临空经济门户的定位，加快构建以机场为核心的综合交通体系，大力推进机场主体工程和配套工程建设，高水平推进航空物流规划和一体化大通关体系建设。加快推进国际空港新城建设，夯实承载基础，搭建功能平台，促进中国电信“云锦天府”信息枢纽等项目开工建设，大力发展现代物流、跨境电商和临空型总部经济。推动龙泉山城市森林公园、简阳城区、简州新城、淮州新城建设，加快东进区域发展。

三是持续提升综合交通能力建设。加快天府国际机场建设，完成双流国际机场扩能改造，开展低空空域管理改革试点，加快建设金堂通用航空机场。建成并开通成蒲铁路，加快成贵、成兰等铁路项目建设，加快推进成都至自贡、成都至西宁等铁路项目的前期工作。继续加快市域铁路公交化运营改造，加密成绵乐城际动车。推动轨道交通加速成网，开通运营地铁1号线三期、3号线二三期，轨道交通运营里程达210公里。开工建设地铁10号线三期、13号线一期和18号线三期。完成成彭高速扩容改造，加快推进天府国际机场高速、三绕高速建设。加快市域快速路网规划建设，开工建设龙泉山旅游快速路，加快推进天邛高速公路、成温邛高速公路扩容改造等项目。积极推进公交都市建设，科学布局、持续优化公交线网，优化完善慢行交通体系，继续打通断头路。

四是大力推进城市基础设施建设。扎实推进中心城区公共服务设施“三年攻坚”行动，加快构建公共服务设施网络体系，新开工公共服务配套项目800个以上。抓好李家岩水库等重大项目建设，加强海绵城市和地下综合管廊建设，加大地下空间综合开发利用。加快建设新一代信息基础设施，提前进行5G网络高标准建设规划及试点，持续提升千兆城市网络能力，加快信息进村入户。加强物联网、大数据、云计算、人工智能等技术综合应用，打造新型智慧城市。开展建筑师负责制试点。对城乡基本公共服务实行清单管理。

五是提高城市精细化管理水平。按照“安全、清洁、有序、方便”目标，深入实施社区发展治理“五大行动”。深化城市管理市场化改革。继续改造棚户区和老旧院落，开展老旧小区加装电梯试点，加快解决拆迁超期安置问题。抓好“两拆一增”行动，实施城市公共空间塑造计划，大力整治背街小巷，打造特色精品街区。继续推进城郊结合部环境治理攻坚行动和百村容貌整治。高标准规划建设城市夜景体系，全域开展最美街道、最差街道评选。深入推进“厕所革命”，新建改建公厕700座以上，消灭农村公共旱厕。深入实施“治堵十条”，完善“11+2”交管模式和交管体制。采取务实举措，继续加大对环卫工人等城市一线管理服务人员关心关爱力度。规范提升共享单车运营服务，大力支持共享交通发展。

（六）大力推动生态环境改善。牢固树立绿水青山就是金山银山的理念，以攻坚精神防治污染、保护生态，扎实推进国家低碳城市建设，进一步巩固提升环境质量，努力让天更蓝、地更绿、水更清，让美丽城镇和美丽乡村交相辉映、美丽山川和美丽人居有机融合。

一是推进绿色低碳转型。构建市场导向的绿色低碳创新体系和产业体系，培育新能源、新材料、智能制造、新能源汽车、节能环保等产业。加快能源消费结构调整，大力实施新能源替代，完善清洁能源基础设施建设，将电动汽车充电桩建设纳入城市社区改造和新楼盘规划。扎实推进装配式建筑示范城市建设，全面推行绿色建筑。推进绿色低碳消费革命，实施成都节水行动，推进分质供水、中水回用。增强绿色供给、推进低碳包装、开展循环利用。鼓励低碳出行，新增公交、公务用车使用新能源车比例分别不低于30%、50%。深化绿色低碳示范单位创建。倡导简约适度、绿色低碳的生活方式，反对奢侈浪费和不合理消费。

二是大力解决突出环境问题。深入实施“治霾十条”和大气“650”工程，淘汰全域大蒸吨燃煤锅炉，强力推进老旧燃气锅炉低氮燃烧改造，提高城市道路扬尘精细化管控水平，严格高排放非道路移动机械区域管控，强化柴油货车排放达标监管，严厉打击生产、销售、使用非标车用燃料行为，强化露天焚烧执法检查，推进科技治霾。深入实施“治水十条”和水“626”工程，深化河长制、推行湖长制，突出岷江、沱江流域水环境治理，加强农业面源污染防治，实施良好水体保护、饮用水环境安全保障工程和城镇污水处理厂提标工程，完成120段黑臭水体综合治理。深入实施“治土十条”和土壤“620”工程，开展受污染场地治理修复试点示范，强化畜禽养殖污染治理，实施化肥农药使用量零增长行动和种养结合循环利用项目。开展垃圾分类试点提质升级行动，推进大林、邓双等6个环保发电厂加快建设。实施固体废弃物防治三年行动攻坚，提升危险废弃物处理能力，加强建筑垃圾再生利用。开展城市噪声污染、光污染等综合整治。突出加强污染防治科研攻关和成果推广应用。坚决打好污染防治攻坚战！

三是加大生态系统保护力度。统筹推进山水林田湖系统治理，提升生态系统质量和稳定性。严守城市开发边界、永久基本农田和生态保护红线，科学评估区域资源环境承载力，探索形成环境空间约束管理办法和产业发展区域环境准入“负面清单”。积极构建生物多样性保护网络。持续推进全域增绿行动，深入实施“增绿十条”，加快建设龙泉山城市森林公园和天府绿道，打造城市生态、人文、经济功能复合体。继续开展城区绿化“春季秋季战役”，大力推进建筑墙体、楼顶、阳台和道桥等城市立体绿化工作，开展二环路增绿提质工程，构建生态区、绿带、公园、小游园、微绿地五级城市绿化体系。大力实施天然林资源保护和退耕还林等重大生态建设工程。开展“川西林盘”保护利用。努力建设好国家森林城市，力争森林覆盖率达到39.5%。积极争创国家生态园林城市。

四是改革生态环境监管体制。持续整改中央、省环保督察反馈问题，加强市级环保督察。推进环保机构监测监察执法垂管体制改革。建立生态文明建设目标评价和考核体系。推动落实生态环境损害赔偿制度改革，建立健全环保信用评价、信息强制性披露、公众参与等制度。做好第二次全国污染源普查和排污许可证核发工作。完善生态补偿激励机制和资源有偿使用制度，探索推行碳排放权和用能权、排污权交易制度，建立健全合同能源管理、环境污染等第三方治理市场。加快建设西部碳交易中心。开展大熊猫国家公园体制试点。

（七）切实提高双向开放水平。深度融入“一带一路”建设，加快建设国家内陆开放型经济高地和国家向西向南开放门户枢纽。

一是加快对外通道和开放载体建设。完善国际航线网络，新增3条以上国际（地区）直飞航线。稳量开行国际铁路班列，优化“蓉欧快铁”网络体系及站点布局，扩大班列覆盖范围，提升运营质量和服务水平。加快建设成都国际铁路港，推进临港产业生态圈建设，打造欧洲产业城和临港经济示范区。争取设立高新西园综保区和天府新区保税物流中心（B型），建成航空肉类、铁路粮食指定口岸，大力推行国际贸易“单一窗口”和空港口岸“7×24小时”通关服务。积极争创国家级“市场采购贸易方式试点”。深入推进国别合作园区建设，打造“一带一路”经济联络基地。

二是加快推进自由贸易试验区建设。深化自由贸易试验区发展三年行动计划，探索贸易便利化、投资自由化、金融国际化等体制机制。加大外资项目招引力度，全面实行“备案+负面清单”管理模式，进一步放宽外资准入。加快自由贸易试验区金融创新，争取跨国公司及央企结算中心落地。发挥国际航空港和国际铁路港的聚合效应，积极探索建设内陆自由贸易港。

三是提高对外交流合作水平。深化与“一带一路”沿线国家经济贸易、人文旅游等合作，鼓励和引导企业“走出去”。完善“3+N”境内外招商网络，新签约引进重大产业项目380个以上，利用外资实际到位110亿美元、到位内资5600亿元。充分发挥中国—欧洲中心的对欧合作功能。积极举办“一带一路”世界文化名城高峰论坛、乒乓球网球比赛等重大国际会议和活动。深入实施外籍人士“家在成都”工程。推动更多国家在蓉设立领事机构，推动相关国际组织在蓉设立办事机构。发展更多国际友城和友好合作关系城市，让成都的“朋友圈”越来越大。

四是积极推动区域协同发展。加快建设成德绵协同创新共同体，加强成都平原经济区城市合作，推动铁路公交化改造运营。加强与泸州、宜宾跨域港口合作。推进区域合作园区建设，加大产业省内转移协同发展工作力度。依托西成客专、成渝客专推动成渝城市群、关中城市群互动发展，深化与西宁、昆明、贵阳、南宁等城市合作。加强与长三角、泛珠三角、京津冀等地区交流合作。

（八）全面加强天府文化建设。紧紧围绕社会主义核心价值观，发展创新创造、优雅时尚、乐观包容、友善公益的天府文化，增强城市文化软实力和文化竞争力，让人文成都别样精彩。

一是繁荣公共文化事业。开展天府文化主题重大历史题材和现实题材文艺创作，推动“一带一路”题材的舞台艺术精品创作，打造《乐舞三国》剧目，创办“天府戏剧节”。开展天府文化“九进”等文化惠民活动，全面完成乡镇（街道）、村（社区）综合性文化服务中心建设，提高基本公共文化服务均等化水平。加强科学普及，倡导全民阅读，建设书香成都。加快推进成都自然博物馆等重大项目建设，高标准建设历史名人文化传承创新工程。持续推进中国足球试点城市建设，开工建设国际足球中心和综合体育馆。积极开展天府绿道、龙泉山城市森林公园系列户外活动，推动全民健身，让市民身体健康、精神愉悦。

二是壮大文化创意产业。加快发展创意设计、数字娱乐、科幻、影视、演艺等文创产业，培育主营业务收入超过50亿元龙头企业6家以上。推动东郊记忆、安仁文创文博等集聚区转型升级，推进熊猫星球、少城国际文创硅谷、临邛文创产业区等重点项目，文创产业增加值实现1000亿元。加快音乐之都建设。提升成都“蓉城之秋”成都国际音乐季、金芙蓉奖等品牌影响力。引进知名音乐企业和重点项目10个以上，举办成都国际音乐（演艺）设施设备博览会，加快建设321梵木创艺区等音乐产业园区和安仁、街子、白鹿、平乐等音乐小镇，建成运营城市音乐厅、露天音乐公园，让音乐浸润城市，让文化滋养心田。

三是保护历史文化遗产。编制《历史文化名城保护规划》。深化不同历史时期城市风貌成片保护，加强古蜀文化、三国文化和历史街区、名人故里、古镇、古建筑和现代工业遗迹等文化遗产的保护利用。打造东华门、邛窑等5大遗址公园，大力推进古蜀文化遗址、“蜀道”申报世界文化遗产。办好中国成都非物质文化遗产节。加大川剧、蜀锦、蜀绣、漆器、皮影等非物质文化遗产保护力度，加快文殊坊、新都锦门等特色产业集聚区发展，加强活态传承、活化保护，让“土玩意儿”更具国际范。

四是拓展对外文化交流。举办第二届中国—中东欧国家艺术合作论坛等国家对外文化交流活动，办好国际女子乒乓球世界杯、ATP250成都网球公开赛等重大体育赛事，筹办2019世界警察和消防员运动会。推进摩洛哥中国文化中心建成开放，加快建成欧亚文化交往中心，培育对外文化交流品牌“天府文化周” ，提升城市文化国际影响力，讲好成都故事，传播成都声音。

（九）突出发展民生社会事业。要抓住人民最关心、最直接、最现实的利益问题，既量力而行，又尽力而为，解决好群众的操心事、烦心事，建设幸福蓉城。

一是继续做好精准扶贫工作。实施脱贫攻坚三年巩固提升行动，继续深化对已脱贫贫困村、贫困人口的“回头看”“回头帮”，开展高标准扶贫开发。加强对口支援甘孜、阿坝深度贫困县精准脱贫工作。继续支持九寨沟灾后恢复重建。

二是优先发展教育事业。建设中小学、幼儿园100所，新增学位10万个。做好婴幼儿照护和儿童早期教育服务。实施优质学校培育计划，努力解决“择校热”“大班额”等突出问题，切实减轻中小学生及家长负担。推进中考制度改革，加快普通高中优质特色发展，大力发展现代职业教育，深入推进“技能成都”建设。支持在蓉高校发展，实施“双一流”大学共建工程，高水平办好成都学院等市属高校，建好中国—东盟艺术学院。筹建“一带一路”高校教育联盟和教育协同创新研究中心。办好继续教育、社区教育和特殊教育等，支持在线教育发展。

三是提高就业和社会保障水平。加强大学毕业生、返乡农民工、关闭破产企业职工等重点群体就业创业援助工作。深化收入分配制度改革，完善企业工资指导线制度。持续推动法定人群参保全覆盖，制定我市对接养老保险金全国统筹实施办法，逐步提高城乡医保筹资标准和实际报销比例，推进医保支付方式改革。健全农村留守儿童和妇女、老年人、残疾康复等关爱服务体系。加强社会救助、社会福利、优抚安置等工作，推进社会关爱援助和慈善事业加快发展。

四是深入推进健康成都战略。加快国家健康城市试点建设，加强重大疾病综合防治和慢性非传染病综合防控，提升中医药、产科、儿科服务能力。实施基层医疗卫生能力提升行动，健全分级诊疗制度，加强医联体内涵建设，加快推进家庭医生签约。强化重大疾病用药、卫生应急药品、低价药的供应保障，加强药品安全监管。巩固公立医院取消药品加成成果。鼓励支持社会办医。深入实施社区养老院建设三年行动计划，深化长期照护保险制度试点。强化粮食安全建设，积极创建国家食品安全示范城市，保障“菜篮子”安全，加大食品安全监管，努力让人民吃得更放心。

五是不断创新社会治理。加强社区发展治理，推进网格化管理与城市公共管理融合，设立社区发展治理专项资金，建成社区综合体45个，积极构建社区生活圈。开展国家安全发展示范城市创建。深化平安成都建设，建立健全城市安全发展体系，压实安全生产监管和主体责任。加快建设国家级危险化学品应急救援基地。改革社会组织管理制度，培育发展行业协会商会、公益慈善等社会组织。依法管理民族宗教事务。妥善做好信访、仲裁、人民调解、社区矫正等工作，有效化解各类风险。加快推进国家防震减灾示范城市建设。加强国防动员工作和后备力量建设，深入开展双拥共建活动。

不驰于空想，不骛于虚声。我们将以造福全体市民为最大政绩，一年接着一年干，一件事情接着一件事情办，集中抓好今年的十大民生实事，努力向全体市民交上满意的答卷！

四、全面建设新时代人民满意政府

打铁必须自身硬。我们要坚持以人民为中心，牢固树立“四个意识”和“四个自信”，认真开展“不忘初心、牢记使命”主题教育，始终以优良作风、严明纪律、过硬本领履职尽责，让党放心，让人民满意！

（一）建设法治政府。认真贯彻市委决策部署，严格执行市人大及其常委会决议决定，主动接受法律监督和工作监督，自觉接受市政协民主监督，全力办好人大代表和政协委员建议、提案，自觉接受民主党派、人民群众和社会舆论监督。完善法治政府建设制度体系，健全行政立法协商机制，抓好重点领域行政立法。严格依法行政，健全重大行政决策程序、“三重一大”事项决策机制，完善重大事项公众参与、专家论证等制度，推进合法性审查全覆盖。继续推进相对集中行政许可权和综合行政执法改革，严格规范公正文明执法。加强行政复议、行政应诉和行政赔偿工作。依法做好统计工作，确保统计数据真实可靠。

（二）建设服务政府。深入推进“放管服”改革，强化事中事后监管，营造公平竞争发展环境。坚持推行市场准入负面清单制度，完善政府权责清单。巩固减证便民成果。深化“仅跑一次”改革。继续实行错时延时工作制试点，更好提供政务服务、开展市场监管、加强城市管理，努力做到群众在哪里服务就在哪里、市场在哪里监管就在哪里。切实加大“云、网、端、数”四位一体的政务云平台建设力度，建立市和区（市）县智慧治理中心，建成全市智慧城市运行中心，提升政务信息资源开放共享应用水平。深入推进网络理政，大力实施“一号申请、一窗受理、一网通办”，建设一体化自助服务系统，推进线上线下融合发展，打造“身边实时政府”。

（三）建设高效政府。建立高质量发展指标体系。深化行政体制改革，统筹使用各类编制资源，科学配置行政部门及内设机构权力、明确职责，推进政事分开、事企分开、管办分离。着力提升公职人员专业化水平和履职能力。加强政府新型智库建设，更好服务政府科学决策。强化政务调研，下深水、察实情，到企业、社区和农村，了解群众诉求、民生短板、发展难题，拿出切实可行的举措解决具体问题。要以“全面落实年”为主线，在抓落实上动真碰硬，以真抓的实劲、敢抓的狠劲、善抓的巧劲、常抓的韧劲，分轻重缓急一锤接着一锤敲，让各项决策部署落地变现。强化行政绩效考核，创新督查督办方式，加强目标、过程、痕迹管理，推进政府管理服务和基层政务公开标准化规范化，广泛推行第三方评估。

（四）建设廉洁政府。深入推进政府系统全面从严治党工作，严格落实主体责任。认真贯彻落实中央八项规定实施细则精神和省市实施细则要求，持续纠正“四风”，特别是形式主义、官僚主义的新表现。坚决查处腐败问题和不正之风，加大微腐败治理力度，加强警示教育，抓早抓小、防微杜渐。加强重点领域和关键环节审计，加强公共资金、国有资产、国有资源、政府项目、公共资源交易等廉政风险点防控。整肃庸政懒政怠政行为，完善干部评价机制，健全正向激励和容错纠错机制，打造一支忠诚干净担当的公务员队伍。

各位代表！核心凝聚力量，思想照亮前方。让我们更加紧密团结在以习近平同志为核心的党中央周围，以习近平新时代中国特色社会主义思想为指导，在党中央国务院、省委省政府和市委坚强领导下，紧紧依靠全市人民，不忘初心、牢记使命，锐意进取、拼搏实干，为持续增进民生福祉、加快建设全面体现新发展理念的城市、实现新时代成都“三步走”战略目标而不懈奋斗！
